# Mortality and morbidity in wild Taiwanese pangolin (*Manis pentadactyla pentadactyla*)

**DOI:** 10.1371/journal.pone.0198230

**Published:** 2019-02-06

**Authors:** Nick Ching-Min Sun, Bharti Arora, Jing-Shiun Lin, Wen-Chi Lin, Meng-Jou Chi, Chen-Chih Chen, Curtis Jai-Chyi Pei

**Affiliations:** 1 Graduate Institute of Bioresources, National Pingtung University of Science and Technology, Pingtung, Taiwan; 2 Department of Natural Resources and Environmental Studies, National Dong Hwa University, Hualien, Taiwan; 3 Institute of Wildlife Conservation, College of Veterinary Medicine, National Pingtung University of Science and Technology, Pingtung, Taiwan; 4 Pingtung Rescue Center for Endangered Wild Animals, National Pingtung University of Science and Technology, Pingtung, Taiwan; US Geological Survey, UNITED STATES

## Abstract

Globally, pangolins are threatened by poaching and illegal trade. Taiwan presents a contrary situation, where the wild pangolin population has stabilized and even begun to increase in the last two decades. This paper illustrates the factors responsible for causing mortality and morbidity in the wild Taiwanese pangolin (*Manis pentadactyla pentadactyla*) based on radio-tracking data of wild pangolins and records of sick or injured pangolins admitted to a Taiwanese wildlife rehabilitation center. Despite being proficient burrowers, results from radio-tracking show that Taiwanese pangolins are highly susceptible to getting trapped in tree hollows or ground burrows. Data from Pingtung Rescue Center for Endangered Wild Animals showed that trauma (73.0%) was the major reason for morbidity in the Taiwanese pangolin with trauma from gin traps being the leading cause (77.8%), especially during the dry season, followed by tail injuries caused by dog attacks (20.4%). Despite these threats, Taiwan has had substantial success in rehabilitating and releasing injured pangolins, primarily due to the close collaboration of Taiwanese wildlife rehabilitation centers over the last twenty years.

## Introduction

The Taiwanese pangolin (*Manis pentadactyla pentadactyla*) is a subspecies of Chinese pangolin found only on the sub-tropical island Taiwan [1]. Taiwanese pangolins live primarily in agricultural fields on mountain slopes below 1,000m a.s.l., with the highest densities of individuals being at about 300m [2–3]. They were commonly distributed throughout lowland Taiwan in the late nineteenth century and mid-twentieth century [4–6]. However, pangolins were harvested for the local traditional medicine and game meat markets, as well as for the international leather trade from 1950 to 1970. It has been estimated that as many as 60,000 individuals were harvested annually during this period [6]. This over-exploitation subsequently caused the population to collapse island-wide, making harvesting economically unviable.

Commercial harvesting and export were banned by the government in the mid 1970s [3,7] and relief from commercial hunting pressure likely led to an increase in pangolin populations in Taiwan [7–8]. However, although this measure ended hunting for the international trade, the demand from domestic markets continued. Over 2,000 individuals annually were estimated to have been sold illegally in game restaurants during the mid 1980s, where the price for a live pangolin could be as high as USD 300 [8].

It was not until the Taiwanese government established the Wildlife Conservation Act in 1989 that domestic consumption was significantly reduced. As a result, based on reports from wildlife rehabilitation centers and burrow surveys, an increase in pangolin numbers has been observed in many locations throughout Taiwan in recent decades [9–11]. This is in contrast to other pangolin populations worldwide for which extirpation, mainly caused by intensive poaching for the illegal trade, continues [12–15].

**Fig 1:**
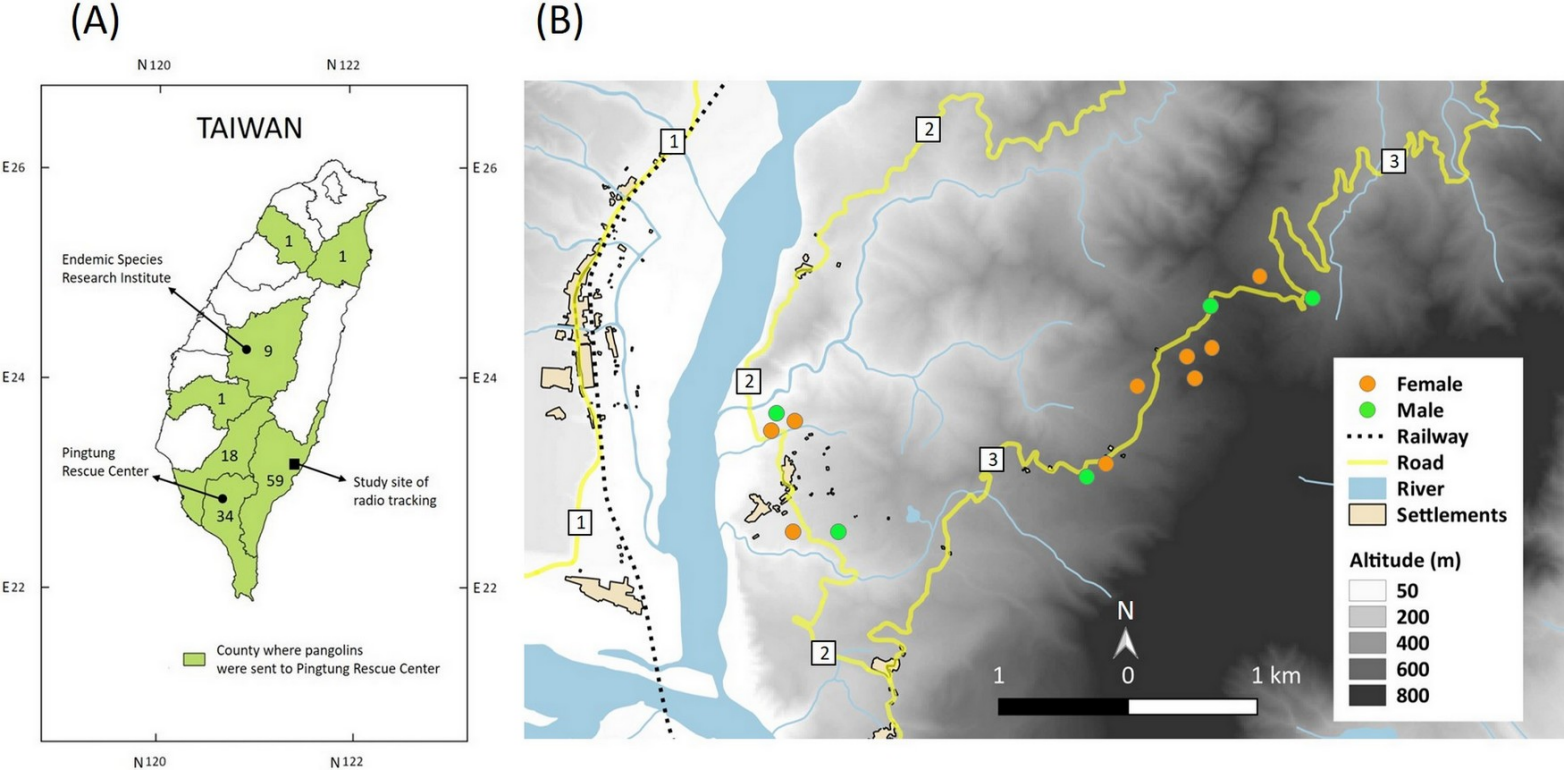
(A) Locations of field study sites for pangolin radio-tracking; the locations of two wildlife rehabilitation facilities, Endangered Species Research Institute and Pingtung Rescue Centre (PTRC); areas in green indicate the county from where pangolins were brought to PTRC from 2006 to 2017, whilst the numbers refer to the number of pangolins brought from that county. (B) Map of the pangolin radio-tracking study site showing the locations of the 14 incidents of dead or injured pangolins. Road No. 1 is a 11m wide paved road with busy traffic (approximately 5,500 cars and motorcycle passing every day; source: Directorate General of Highways, MOTC. https://www.thb.gov.tw/); Road No. 2 is a 7m wide paved road with much lighter traffic; Road No. 3 is a 7m wide paved country road with little traffic.

Besides poaching, there have also been other factors that have affected the mortality of pangolins in Taiwan. From 1993 to 2009, a total of 117 wild Taiwanese pangolins were brought to the wildlife rehabilitation facility of the Endemic Species Research Institute (ESRI) in central Taiwan (Fig 1A). 82.9% of them required serious medical care upon arrival. Records showed that 50% of this group were injured due to gin (or leg-hold) traps, whilst 23% were malnourished. Animal attacks and vehicular collision also caused serious trauma in some cases [10]. Gin traps have been commonly used by farmers in Taiwan for pest control and to hunt small mammals. Due to the high incidence of non-target animals being killed, including endangered wildlife, the manufacture, sale and use of gin traps was finally banned under the Animal Protection Act of 2011.

Additionally, the results from pathological analysis of dead pangolins found pneumonia and gastric ulcers to be the primary causes of mortality in wild pangolins from northern Taiwan [16]. Recently, Khatri-Chhetri et al. [17] found a high prevalence of hepatic and respiratory lesions in dead pangolins from southeastern Taiwan. They proposed this might be a result of long-term exposure to toxins in the environment, due to pesticides and herbicides commonly used on Taiwanese farms.

In general, although poaching is no longer a significant threat to the Taiwanese pangolin population, other anthropogenic threats continue to impede its recovery. We present two sets of information that illustrate the leading causes of mortality and morbidity in this species in the wild: one data set comes from a long-term radio-tracking project in Taitung, southeastern Taiwan, from 2009 to 2017; the second data set is an analysis of pangolin admission data from the Pingtung Rescue Center for Endangered Wild Animals (PTRC) from 2006 to 2017.

## Methodology

### Ethics statement

Ethics approval was granted by the Laboratory Animal Center, National Pingtung University of Science and Technology (NPUST). Pangolins were captured live for radio-tracking with permission granted by the Taiwan Forestry Bureau (permit numbers 0980129850, 0991616024, 1011701139, 1031700176, and 1050143346) as required by the Wildlife Conservation Act, 2013. Anesthesia and blood sampling were carried out following procedures described in Khatri-Chhetri et al. [18]. This research is part of the “Pangolin biology and ecology project”, a long-term study conducted by the Institute of Wildlife Conservation, NPUST, since 2009.

### Radio-tracking

Radio-tracking was conducted in the southern part of the isolated Coastal Mountain Range, Taitung County, southeastern Taiwan (Fig 1A). It is one of the areas in Taiwan where a stable pangolin population can be found. Pangolin density here is estimated to be 12.8 ind./100ha [19]. The study area is approximately 1,000ha in size with an elevation range between 100m and 700m a.s.l. The climate is tropical with a hot, wet season from April to November and a cooler, drier season from December to March. Due to a long history of human encroachment, there is no primary forest in the study area. The landscape is highly fragmented, interspersed with secondary forest, managed tree plantations, bamboo forest, grassland and agricultural land.

From 2009 to 2017, a total of 47 pangolins from this area were captured and radio-tagged. Three models of radio transmitters were used: Telonics MOD-125 (53g with active mode; 932 E. Impala Ave. Mesa, Arizona, USA), ATS R2030 and ATS R2020 (24g and 12g with active mode respectively; ATS, Inc. PO Box 398 470 First Ave. N. Isanti, Minnesota 55040). Transmitter selection was dependent on the body weight of the pangolin tagged but the weight of the transmitter was < 1.5% of body weight in all cases. The radio transmitter was fixed on the non-vascularized portion of a large and thick scale on the rump using pan head screws (stainless steel, 2.4mm, 8mm in length) and hex nuts (stainless steel, 5.5mm in external diameter, 2.4mm in internal diameter). In order to minimize the possibility of detachment when the transmitter was pulled or dragged while the pangolin was moving through dense vegetation or a burrow, a piece of hose pipe rubber or sling belt was attached between the transmitter and scale.

After being released back into the wild, pangolins were radio-tracked daily for 7 days, usually every other week, mainly at night, using a telemetry receiver (TR4; Telonics) and a directional H-antenna (RA-2AK or RA-23K) to confirm their locations and activity status. Intervals between continuous tracking periods rarely exceeded 2 weeks. The transmitter was replaced when battery power was running low to ensure monitoring was continuous.

All individuals were assigned to one of three age groups according to their body weight. For both sexes, animals weighing less than 1.5kg were classified as juveniles. Males were classified as subadults if they weighed between 1.5kg and 4kg, while males weighing more than 4kg were classified as adults. Females were classified as subadults if they weighed between 1.5kg and 3kg, while females weighing more than 3kg were classified as adults. Based on long-term monitoring of radio-tagged individuals and their offspring in the study area [20], Taiwanese pangolins become independent at about 6 months [21], weighing approximately 1.5kg when they leave their mother, and reach their adult body weight at about 3 years old.

Among the 47 radio-tracked individuals, 20 were adults and 27 were subadults when tagged. A total of 25 females and 20 males were recorded, with two animals missing sex information. Unexpected incidents occurred for 22 animals during the study–transmitter signal was lost and never recovered for 9 animals; 13 animals were involved in 14 incidents, where the animal was found dead or in danger by the research team (Fig 1B). These cases provide insight into the causes of Taiwanese pangolin morbidity and mortality in the wild.

### Rehabilitation center data

A total of 131 (58 females, 73 males) wild pangolins were received for rehabilitation by PTRC from members of the public and government officials from 2006 to 2017 (Fig 1A). The majority of the pangolins were from southern Taiwan (83.6%), notably southeastern Taiwan (43.9%). For those individuals with injuries or in poor physical condition, medical checks, treatments and blood collection were conducted upon arrival. For healthy individuals, blood collection and medical checks were done the next day. All pangolins were kept individually in a L1.2m×D1.2m×H2.4m cement chamber with a wooden hide box; food and water were provided *ad libitum*.

Every animal received a complete physical examination from veterinarians. Diagnoses were classified into morbidity categories such as trauma (encompassing limb injuries, tail injuries and collisions), other medical conditions (such as abscessation, diarrhea and emaciation), and unknown or undetermined. Diagnosis of disease or anomalies was based on evidence of infective agents (inflammatory cell aggregates, suppuration and mucopurulent discharge) and results from diagnostic tests such as complete blood count, serum chemistry and total protein assays to determine emaciation [18]. Full body X-rays were conducted to ascertain trauma. Clinical diagnoses with any uncertainty were classified as undetermined or unknown in this study.

As the goal was to release all rehabilitated pangolins back to the wild as soon as possible, deworming and other anti-parasite treatments were not performed as parasites are considered a natural occurrence in wildlife. Prophylactic treatment was only given if long-term care in PTRC was necessary. Healthy pangolins were returned back to the wild within 2 weeks; most were released within a week. Those suffering from injuries or health issues when admitted to PTRC were usually released within 6 months. Pangolins were released in safe environments or protected areas close to where they were found.

Individuals categorized as healthy upon arrival to PTRC were excluded from statistical analysis in the study. Healthy pangolins were sometimes sent to PTRC after being accidentally caught by members of the public, probably due to increased public awareness to bring any wildlife encountered to rehabilitation centers for further assistance.

## Results

### Radio-tracking

Of the 14 cases described (Table 1), there was no difference between the number of subadult females and adult females involved in unexpected incidents (5:4). However, a lot more subadult males were found in life-threatening or life-ending circumstances compared to adult males (4:1). Case 12 and 13 refer to the same individual. With the exception of Case 1 and 2 where information was incomplete, almost all remaining cases (10 cases, 83.3%) happened during the dry season, whilst the other two cases happened very close to the beginning or just after the dry season (October and April, respectively); no cases were recorded at the height of the wet season. The average tracking duration for the known cases was 217.3 days (N = 12, SD = 246.3; range: 45 to 869 days) (Table 1).

**Table 1:** Details of the fourteen cases with unexpected incidents for the radio-tracked Taiwanese Pangolin (*Manis pentadactyla pentadactyla*) from 2009 to 2017.

Case No	Sex	Trap and tag		Died or in danger		Duration of tracking (days)	Description
		Weight (kg)	Age group	Date	Weight (kg)		
1	F	—*	Adult	2009/—/—	> 4.00	< 1 year	It was found dead and stuck in a hollow tree trunk.
2	F	—*	Subadult	2009/—/—	< 3.00	< 1 year	It was found dead and stuck in a hollow tree trunk.
3	F	1.65	Subadult	2010/12/15	—	45	It was found dead on the forest floor; cause of death is unknown.
4	M	2.20	Subadult	2011/04/17	2.50	66	It was found dead in a gin trap by a local person and its body was handed over to the researcher.
5	F	1.90	Subadult	2012/12/24	—	40	The tag was found detached by a sharp object, the pangolin had disappeared.
6	M	2.15	Subadult	2012/12/25	3.80	468	It was captured alive by a local person while crossing the Songlin Road (Fig 1) and handed over to the researcher.
7	F	2.10	Subadult	2013/01/15	2.10	69	It was found dead in a resting burrow; cause of death is unknown.
8	M	3.65	Subadult	2013/02/13	3.00	98	It was found trapped in a gin trap by the researcher, was subsequently rehabilitated and released.
9	M	2.60	Subadult	2014/03/07	—	51	Its tag was found detached by a sharp object; the pangolin had disappeared.
10	F	3.90	Adult	2014/03/10	3.60	367	It was found dead in a collapsed resting burrow.
11	M	2.15	Subadult	2015/02/05	2.10	142	It was found dead in a collapsed resting burrow.
12	F	1.85	Subadult	2015/02/20	4.00	869	Pangolin LF28 was found stuck in a foraging burrow and was rescued.
13	F	4.00	Adult	2015/10/10	4.00	232	LF28 was then seen again missing a left hind limb in a camera trap photo. The injury was most likely caused by a gin trap. According to a later photo, this animal survived.
14	F	2.80	Subadult	2016/02/09	—	160	Its tag was found detached by a sharp device, the pangolin had disappeared.

* missing data.

With the exception of Cases 3 and 7 (Table 1) where causes of death could not be determined, fatalities mostly happened when individuals were stuck in burrows or hollow tree trunks (5 cases, 41.7%). These fatalities involved subadults and adults (3 adults, 2 subadults). Only 1 pangolin (Case 12) was found alive, suggesting a high mortality rate when pangolins get stuck in burrows. There were 3 animals (Case 4, 8, 13) found injured by gin traps, and another 3 animals (Case 5, 9, 14) had their radio tags removed by non-natural force, most likely by local people during poaching. Though pangolins continue to be poached, local people may catch them under other circumstances and hand them over to researchers (e.g. Case 4, Case 6) or wildlife rehabilitation centers.

### Rehabilitation center data

**Fig 2:**
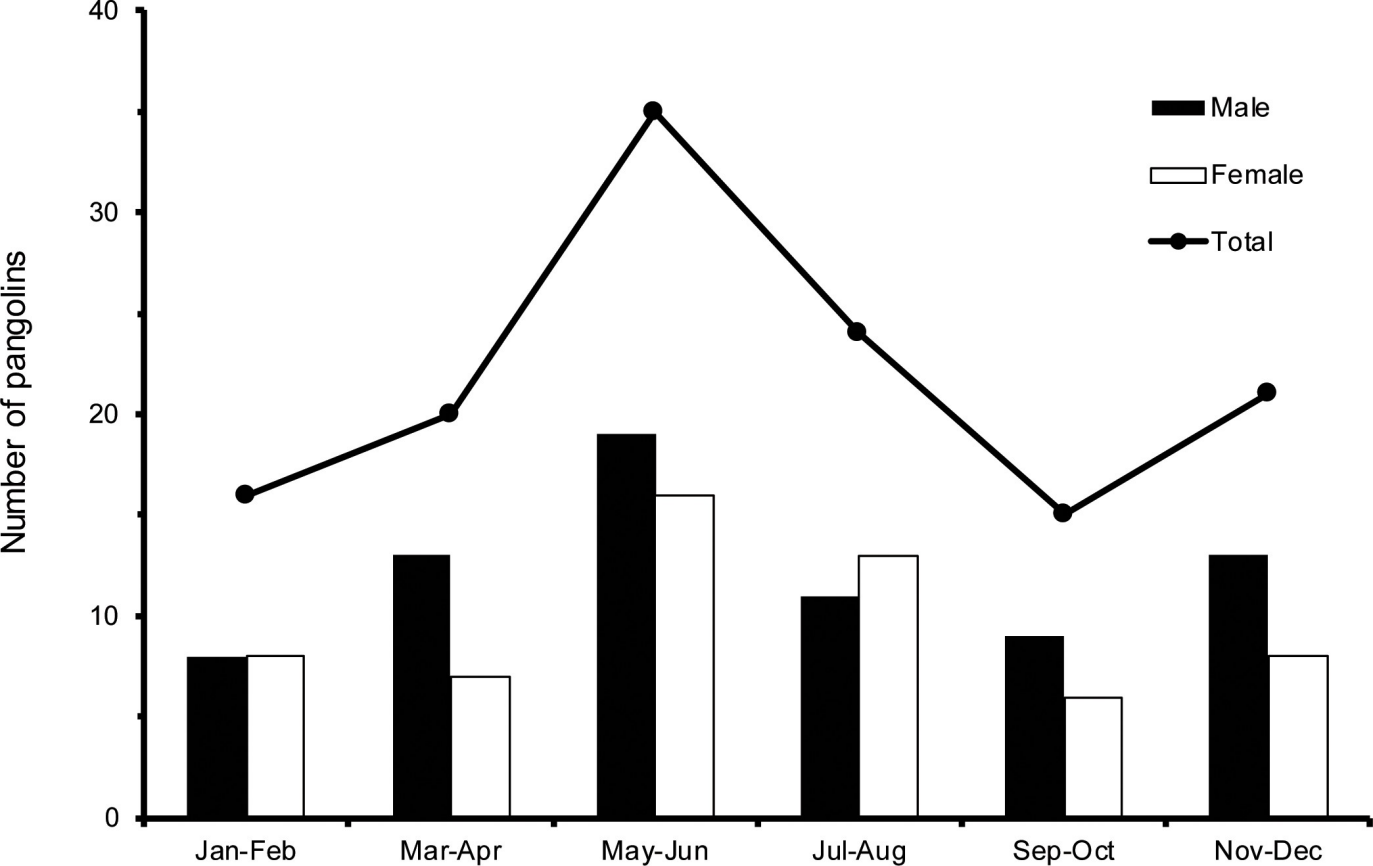
Bi-monthly totals of Taiwanese Pangolin admitted to Pingtung rescue center from 2006 to 2017.

Our records display that the sex ratio for pangolins admitted was male-biased (F:M = 44.3:55.7), comprising mainly subadults and adults (Table 2). The number of pangolins admitted varied significantly in different months (X^2^ = 25.72, df = 1, p < 0.005), with highest numbers from May to August, followed by November to December, with both sexes showing the same seasonal pattern (Fig 2). Among the 131 individuals admitted, 57 (43.5%; 31 females, 26 males) were healthy, only requiring care to recover from the stress of capture.

**Table 2:** Sex and age-groups of the Taiwanese pangolin (*Manis pentadactyla pentadactyla*) brought to the PTRC from 2006 to 2017.

Age groups	Female		Male		Sum	
	n	%	n	%	n	%
Juvenile	8	13.8	4	5.5	12	9.2
Subadult	29	50.0	39	53.4	68	51.9
Adult	21	36.2	30	41.1	51	38.9
Total	58	100.0	73	100.0	131	100.0

Compared with the sex ratio of the total number of admitted individuals, there were significantly more males (63.5%) in the morbid group (X^2^ = 0.182, df = 1, p > 0.1). There was no difference in age-group distribution for females (X^2^ = 0.613, df = 2, p > 0.1) and males (X^2^ = 0.778, df = 2, p > 0.1) across the total or morbid group, suggesting that morbidity is not age-dependent. Trauma (73.0%) was the most prominent reason for morbidity (Table 3). Cases of trauma involving limb injuries (77.8%) were all caused by gin traps, while those involving tail injuries (20.4%) were most likely caused by dog bites; one case of trauma was due to vehicular collision. The remaining cases involved other medical conditions including emaciation, diarrhea and abscessation (Table 3). There was no sex difference in the distribution of types of morbidity (X^2^ = 0.44, df = 3, p > 0.1).

**Table 3:** Causes of morbidity in Taiwanese pangolins (*Manis pentadactyla pentadactyla*) brought to PTRC from 2006 to 2017.

Morbid	Female	Male	Sum
Trauma	20	34	54
* Limb injury*	*14*	*28*	*42*
* Tail injury*	*6*	*5*	*11*
* Collisions*	*0*	*1*	*1*
Other medical conditions	4	10	14
Injury data missing	3	3	6
Total	27	47	74

The majority of the pangolins received by PTRC were released back into the wild after rehabilitation; one-tenth of the animals died after arrival (Table 4). Among the 14 individuals that died in PTRC, all except 2 belonged to the trauma group (11 Limb injury, 1 Collisions). There was also no sex difference in rehabilitation outcomes (X^2^ = 0.52, df = 2, p > 0.1).

**Table 4:** Outcomes for Taiwanese pangolins that were admitted to PTRC from 2006 to 2017.

	Female		Male		Sum	
	n	%	N	%	n	%
Released into wild	53	91.4	63	86.3	116	88.5
Long-term captive care	0	0.0	1	1.4	1	0.8
Died	5	8.6	9	12.3	14	10.7
Total	58		73		131	

## Discussion

From the two data sets presented herein, it is obvious that the continued use of gin traps is the most significant threat to the Taiwanese pangolin. Gin trap related injuries and mortality comprised more than half (56.8%) of morbidity cases in PTRC records (Table 3) and 25.0% in radio-tracking cases (Table 1). Previous data from ESRI in central Taiwan also revealed a similar pattern: 61.8% of morbid pangolins received had been injured by gin traps [10]. Though the production, sale and use of gin traps has been banned in Taiwan since 2011, a recent survey in rural Taiwan found 23.1% (= 9/39) of hardware stores interviewed still offered gin traps for sale to farmers [22]. Gin traps are still commonly used for pest control in agricultural areas and remain a major threat to the endangered Leopard Cat (*Prionailurus bengalensis*) in rural Taiwan [23–24]. Although strengthening law enforcement is urgently required, providing farmers with animal friendly techniques to replace current inhumane pest control devices should also be mandatory.

Our data obtained from radio-tracking wild pangolins has uncovered surprising findings that were not reflected in the PTRC records: pangolins have a high risk of mortality whilst engaging in daily activities such as hunting for food or maintaining resting burrows. Pangolins being fossorial in nature, are widely assumed to be excellent diggers. For this reason, they are called *Chuan-Shan-Jia* (meaning “Excavation-Mountain-Armor”) in Chinese. However, this study has shown that getting trapped in tree hollows or underground burrows could be a significant risk factor for mortality in pangolins. Such incidents were more frequent during the dry season (Table 1) when digging for termites was necessary due to the scarcity of ants, the primary source of food for pangolins [25–29]. It could be surmised that the paucity of food during the dry season caused a loss in body weight [20], resulting in poorer body condition, further compromising an animal’s ability to avoid such dangers. Lin [27] reported that the average length of a feeding burrow was 120.1±69.5cm, while the length of a resting burrow could reach 201.6±94.8cm. Furthermore, the mosaic of land uses of southeastern Taiwan is dominated by various agricultural practices that are incompatible with the natural landscape. Such practices affect landforms and soil properties, possibly increasing the chances of burrows collapsing causing pangolins to get trapped and die. Such fatal outcomes could pose a significant threat to the local population [30].

Although the number of dog attacks was comparatively few, their impact cannot be overlooked. *Canis familaris* outnumbers all canids combined and has affected various wildlife communities due to predation [31–32]. Dogs pose a serious threat to wildlife in ways other than bites. Lenth et al. [33] observed how the intrusion of dogs exploring new territories restricted the movement of various wildlife, in addition to causing a temporal shift in patterns of activity. Therefore, dogs might also indirectly affect the movement of pangolins, making them more visible to other predators such as humans. For example, pangolins might change their routes to avoid free ranging dogs, forcing them to pass through areas such as farmland that contain trapping devices.

Moreover, dogs carry ticks, in particular those belonging to the Ixodidae family. This family of ticks is known to affect pangolins as well [34–35]. Ticks, especially in their nymph stage, can be a serious threat to the health of medium and large mammals due to the various pathogens they carry [36]. Due to their sensitive immune system [37], pangolins can be especially vulnerable to ticks. Ticks often attach themselves under scales, causing infections [35–36] in pangolins as the ticks complete their life cycle.

Other medical conditions resulting in mortality and morbidity in the Taiwanese pangolin include diarrhea, emaciation and abscessation. Autopsy records revealed that gastric ulcers are common in this species [16–17] and might act as a contributing factor for diarrhea. Furthermore, a depletion of food resources due to human activity may cause emaciation and stress in the Taiwanese pangolin, conditions which are also correlated to gastric ulcers.

Subadults are especially vulnerable to injury during the summer months from May to August (Fig 2), as the weaning period ends and maternal care stops [21]. This pattern is also consistent with previously reported rehabilitation records [10]. During this time, subadults must find new, unoccupied territory. In the course of finding new territory [38], pangolins might be injured as they pass through areas subjected to anthropogenic disturbances. A higher incidence of injury also occurred during the winter months from November to January (Fig 2), the reproduction season of the Chinese pangolin [39]. In short, pangolins face a greater risk of injury as their activity increases.

The incidence of injury in males is higher than in females in both ESRI and PTRC records, possibly due to the much larger home range of males [27]. Males, being more active than females, are more exposed to encounters with humans and other potential hazards [16]. Similar behaviors in males of other species have been observed as they are brought into wildlife rehabilitation facilities more frequently than females [40–41]. However, it is also plausible that injured females have higher mortality rates in the wild, possibly due to their smaller size and weight, meaning fewer females are found alive and brought to rehabilitation facilities. It might also explain why there were only a few juveniles in rehabilitation center records. However, further research is required to determine if there is a correlation between body weight and pangolin survival rates in the wild.

Despite the negative impact of trapping devices and agricultural practices on the Taiwanese pangolin, one positive outcome is that rehabilitation centers in Taiwan have developed an extensive knowledge sharing network to better protect this cryptic mammal. This joint effort has enhanced understanding and awareness, resulting in a declining rate of mortality for morbid pangolins. Close collaboration between wildlife rehabilitation centers such as ESRI and PTRC, has greatly reduced rates of mortality from 35.9% [10] to 10.7% (Table 4) and boosted release rates from 60.7% [10] to 88.5% (Table 4).

The two sets of data from ESRI and PTRC also reflect how the condition and numbers of pangolins received by wildlife rehabilitation centers have changed over time. Many more healthy pangolins were received by PTRC than ESRI (43.5% versus 17.1%). The difference might be due to an increase in public awareness and popularity of wildlife rehabilitation centers in recent years, with more people willing to hand over pangolins to wildlife rehabilitation centers than before. It might also be due to the increase in the pangolin population and subsequent increase in distribution, where more individuals are now more likely to encounter humans during locomotion. Such data demonstrates the important role wildlife rehabilitation centers play in research and public education, which in turn contribute to the recovery and *in situ* conservation of the Taiwanese pangolin.

For the successful conservation of any endangered species, it is key to understand the factors affecting mortality of that species. In this paper, we described both natural and anthropogenic factors for the loss of the Taiwanese pangolin, some of which represent previously undocumented cases. Despite the numerous threats facing the Taiwanese pangolin, the banning of illegal poaching in Taiwan has enabled the pangolin population to stabilize and even to increase in many areas in recent decades [9–10], contrary to populations in all other range countries. This is strong evidence that poaching for illegal markets alone can cause the extirpation or heighten the endangered status of this species in other countries it inhabits.
