# Correction: Mortality and morbidity in wild Taiwanese pangolin (*Manis pentadactyla pentadactyla*)

**DOI:** 10.1371/journal.pone.0212960

**Published:** 2019-02-21

**Authors:** The PLOS ONE Staff

The corresponding author's name is spelled incorrectly. The publisher apologizes for this error. The correct name is: Kurtis Jai-Chyi Pei.

In the Data Availability statement, there is an error in the email address provided for Dr. Hsiu-Hui Su, the Director of the Institute of Wildlife Conservation. The correct email address is: hhsu@mail.npust.edu.tw.
